# Exposure to Environmentally Relevant Levels of PFAS Causes Metabolic Changes in the Freshwater Amphipod *Austrochiltonia subtenuis*

**DOI:** 10.3390/metabo12111135

**Published:** 2022-11-18

**Authors:** Georgia M. Sinclair, Sara M. Long, Navneet Singh, Timothy L. Coggan, Matthew P. J. Askeland, Oliver A. H. Jones

**Affiliations:** 1 Australian Centre for Research on Separation Science (ACROSS), School of Science, RMIT University, Bundoora West Campus, P.O. Box 71, Bundoora, VIC 3083, Australia; 2 Aquatic Environmental Stress Research Group (AQUEST), School of Science, RMIT University, Bundoora West Campus, P.O. Box 71, Bundoora, VIC 3083, Australia; 3 ADE Consulting Group, Williamstown North, VIC 3016, Australia

**Keywords:** bioconcentration, environment, metabolomics, monitoring, regulation, toxicology, Gas Chromatography, amphipods, water quality

## Abstract

Per and polyfluoroalkyl substances (PFAS) are of concern to environmental regulators due to their widespread occurrence, persistence and reported toxicity. However, little data exist on the effects of PFAS at environmentally relevant concentrations. The development of molecular markers for PFAS exposure would therefore be useful to better understand the environmental risks of these compounds. In this study, we assessed if such markers could be developed using Gas Chromatography–Mass Spectrometry-based metabolomics. We exposed the freshwater amphipod *Austrochiltonia subtenuis* to a range of environmentally relevant concentrations of perfluoro-octane sulfonic acid (PFOS), hexafluoropropylene oxide dimer acid (GenX) and perfluorohexanesulphonic acid (PFHxS) for 7 days at five concentrations. A metabolic response was detected in all concentrations and treatments even though the survival rates only differed significantly at the highest exposure levels. The metabolic response differed between compounds but all three PFAS induced changes in the levels of amino acids, fatty acids, and cholesterol, in line with the literature. PFOS was found to bioaccumulate. Both GenX and PFHxS were eliminated from the amphipods, but PFHxS was eliminated at a slower rate than GenX. This information improves our understanding of the sublethal effects of PFAS as well as their environmental fate and behaviour.

## 1. Introduction

Per and polyfluoroalkyl substances (PFAS) are a diverse group of human-made compounds that have been in use since the 1940s. They are used in a range of products such as Teflon and Scotchgard, food packaging, cosmetics, waterproof textiles and aqueous film-forming foams (AFFFs) [1]. PFAS are very resistant to degradation and are often labelled ‘forever chemicals’. They have been detected in almost every environmental compartment (e.g., soil, sediment, oceans and freshwater) as well as in wildlife and humans throughout the world [2]. 

It was previously assumed that PFAS were inert and little regard was given to the environmental fate and the ecological impacts of these compounds to organisms and environmental health. This was later realized to be a mistake and, today, there are serious concerns about their potential effects, causing growth/developmental delays and cancer [1]. While there is a substantial amount of data on PFAS toxicity, environmental exposure studies on these compounds tend to use substantially higher concentrations of PFAS than are generally found in the environment [3]. As such, it is still unclear what the biological effects of PFAS may be at environmentally relevant exposure levels [3,4]. Effective management of these compounds at the government, local and industrial levels is therefore challenging because it is difficult to prove that environmental harm has occurred. 

Standard ecotoxicology endpoints, such as growth or reproduction, may not register the effects of chronic, low-dose toxicant exposure. However, recent work by Beale et al. [5] showed that environmental toxicological studies can be greatly enhanced when coupled with molecular biology endpoints, such as the use of metabolomics and other omic techniques. Omic techniques include the detection of genes (genomics), mRNA (transcriptomics), and proteins (proteomics) in a biological sample [5]. Metabolomics is the study of small-molecule metabolites such as fats, sugars and amino acids and how these change in response to external factors such as disease, or toxicant exposure [6]. Small changes in the network of metabolites can indicate the level of an organisms’ overall health and a report from the Expert Health Panel for PFAS noted that “better biomarkers of the net effect of all PFAS would be extremely useful” [7]. Finding the range of doses where PFAS concentrations disrupt the biochemistry of biota is therefore likely to enhance our overall understanding of the mode of action, bioaccumulation, and toxicity of PFAS in ecosystems [8,9].

Out of the thousands of potential PFAS, perfluorooctanoic acid (PFOA) and perfluoro-octane sulfonic acid (PFOS) are the most commonly detected in the environment, closely followed by perfluorohexanesulfonic acid (PFHxS). PFOA has already been listed by the European Chemical Agency as a substance of very high concern and, in 2022, UN countries agreed to a global ban of PFHxS, with no exemptions, by adding it to Annex A of the Stockholm Convention on persistent organic pollutants (POPs) [10]. This means the use of both PFOA and PFHxS is now restricted, even though they are still commonly detected in the environment [11]. The phase-out of these compounds has led to the introduction of shorter-chained compounds such as GenX (hexafluoropropylene oxide dimer acid). GenX has recently been detected in ecosystems in Europe and North America, indicating that it may be just as persistent as other PFAS compounds and, as such, it is of increasing concern worldwide [12,13].

This work focused on PFOS, PFHxS and GenX. Three experiments were carried out in controlled laboratory exposures, using the non-model organism *Austrochiltonia subtenuis* (an endemic and common freshwater amphipod in Victoria, Australia) [14]. They are small, usually <1 cm, benthic shredders and are a food source to many fish, birds and invertebrate species. This species was chosen as it is crucial to the function and structure of invertebrate communities. Gas Chromatography–Mass Spectrometry (GC–MS) was used to identify metabolites in amphipods and assess how they changed in response to environmentally relevant levels of PFAS. We were particularly interested to see if a common biochemical response would be evident across compounds. An additional objective was to calculate bioconcentration factors (BCFs) for each compound and use these to help understand the metabolomic data. The overall aim was to contribute to the understanding of the potential effects on PFAS at environmentally relevant exposure levels and, ultimately, inform environmental regulation of these compounds.

## 2. Materials and Methods

### 2.1. Organism

Live amphipods *(Austrochiltonia subtenuis)* were originally collected from a wild population in Deep Creek, Bulla Rd, Bulla, Victoria, Australia (S 37°37.919157′ E 144°47.995837′). An amphipod culture has long been established (>5 years) and maintained in house at RMIT University (Melbourne, Australia). The amphipods are maintained in aquaria using a standard artificial media (SAM) modified from Borgmann [15]. The SAM consisted of reverse osmosis (RO) water (18.2 mΩ), obtained in house, with 0.23 mM NaHCO_3_, 0.061 mM CaCl_2_, 0.032 mM MgSO4, 0.47 mM NaCl, 0.0087 mM KCl, 0.17 mM MgCl_2_ and 0.0009 mM NaBr. Cultures were maintained at 21 ± 1 °C under a 16:8 h light: dark photoperiod. The culture was fed powdered fish food (Tetramin^®^, Tetra Werke, Melle, Germany) and yeast–cerophyll–trout chow (YCT), made in house, every second day.

### 2.2. Chemical and Concentrations 

A perfluoro-octane sulfonic acid (PFOS) standard was purchased from Sigma-Aldrich (Melbourne, VIC, Australia). Hexafluoropropylene oxide dimer acid (common name, GenX) and perfluorohexanesulphonic acid (PFHxS) standards were purchased from Wellington laboratories (Guelph, ON, Canada). Experimental solutions of differing concentrations of each compound were prepared from stock solutions diluted with SAM as necessary.

### 2.3. PFAS Exposure

Environmental standards of PFAS vary around the world. In Australia, the Heads of the Environmental Protection Agency for Australia and New Zealand (HEPA) have set PFAS guideline values based on the precautionary principle. The concentration levels used in this study were based on the PFAS National Environmental Management Plan (NEMP) (Version 2.0, National Chemicals Working Group of the Heads of EPAs Australia and New Zealand, Department of Climate Change, Energy, the Environment and Water, Australian Government), created by HEPA, which suggests a guideline value of 0.13 µg/L of PFOS to protect 95% aquatic species [2]. This was used as the basis of concentrations for all compounds. We exposed amphipods to five concentrations, both above and below the 0.13 µg/L limit (Table 1). This ensured that a) we could be sure of seeing an effect and b) that we could assess differences between high and low exposure levels. Variations in concentrations for each compound were due to small differences in the initial standard concentrations when purchased and the variation introduced during preparation of standards. 

For each study, two days prior to the test, 14-day-old (juvenile) amphipods were collected into 2 L beakers until the experiment from the in-house culture tanks, using a 250 µm sieve. On day one of the test, 20 juvenile amphipods were randomly placed in each 600 mL beaker containing 400 mLs of SAM or PFAS treatment solution, including a substrate of 2 × 2 cm square of gauze cut from a sterilized bandage (Livingstone Triangular bandage—bleached calico) purchased from a local supermarket.

Beakers were randomly placed in an incubator with aeration maintained throughout the test. Amphipods were fed every second day with 1 mL powdered fish food (Tetramin^®^, Tetra Werke, Germany) solution (90 mg of ground Tetramin/50 mLs reversed osmosis water) and 1 mL YCT. The amphipods were exposed to a 16:8 h light: dark photoperiod and the temperature was maintained at 20 °C (±2) throughout the test. Water quality parameters (dissolved oxygen, pH, conductivity, and ammonia) were measured for each treatment at the conclusion of the experiment (Supplementary Materials—Table S1). A solvent control with laboratory-grade methanol at a concentration of 0.25 mL/L was run alongside the exposure treatments (no significant changes in survival or metabolites were detected from the solvent control). Amphipod survival was also recorded in each treatment (Table S2). On day 7, amphipods were counted from each beaker and immediately placed in microcentrifuge tubes on dry ice, to quench metabolites, and stored at −80 °C until analysis. Amphipod tissue was also collected for PFAS analysis to assess bioconcentration. Water samples were collected for PFAS measurement analysis.

### 2.4. Sample Preparation and Metabolite Extraction

A 300 µL aliquot of a methanol:chloroform (9:1) mixture was added to each sample. Amphipods were then homogenized by manual grinding using a metal spatula (which was cleaned between samples) within the microcentrifuge tubes and placed on dry ice. Samples were then sonicated for 15 min in an ultrasonic water bath and then centrifuged in an Eppendorf 5424 Centrifuge (Macquarie Park, NSW, Australia) at 5500 rpm for 15 min at room temperature. The supernatant was decanted into glass inserts and allowed to evaporate in a fume hood. 

Dried extracts were derivatized using 20 µL of methoxyamine in pyridine solution (20 mg/mL). The samples were then vortexed for 30 s and left for 17 h at room temperature. Next, 20 µL of N-methyl-N-(trimethylsilyl)trifluoroacetamide (MSTFA) was added. Samples were vortexed again and left for one hour to react. Analytical-grade hexane was added to each sample (300 µL) prior to GC–MS analysis. A pooled biological quality control (PBQC) was prepared by pooling 7.5 µL of each sample extract, mixed thoroughly and aliquoted into 5 replicates. PBQCs were analysed along with the samples and controls/blanks to assess repeatability, instrument drift and quality control.

### 2.5. Gas Chromatography–Mass Spectrometry 

Samples were analysed in random order. GC–MS analysis was performed using an Agilent 7890B gas chromatograph coupled to an Agilent 5977B mass spectrometer (Agilent, Santa Clara, CA, USA). The gas chromatograph was operated in the splitless mode, with a purge flow at 2 min. Separation was performed with an DB5–MS column (30 m × 250 μm, 0.25 μm) using helium as carrier gas at a flow rate of 1 mL per min. The injection volume was 1 μL and the injector temperature was 250 °C. The oven temperature rose from 35 to 300 °C at 25 °C/min, held at this temperature for 5 min, then increased to 310 °C (at 5 °C/min) and held for 5 min. 

Mass spectra were recorded at 1.5 scans/s over an *m*/*z* range of 35–550. Metabolites were putatively annotated as level 2 compounds (based upon spectral similarity with public/commercial spectral libraries) where possible, using the National Institute of Standards and Technology (NIST MS search 2.0) library. Raw area retention times (RT) from each significant peak that occurred was recorded (Table S3). Environmental metabolomic reporting standards as described by Morrison et al. [16] were adhered to.

### 2.6. PFAS Determination Using Liquid Chromatography—Mass Spectrometry 

PFAS was measured in water and amphipod tissue samples using an in-house method (ESA-P-ORG-16) based on Coggan et al. [17]. The methodology uses a triple quad LC–MS/MS Shimadzu 8060 with a C18 column (3 mm I.D. × 30 mm, P/N number 228-41606-91). The samples for biota were extracted using pure LC–MS-grade methanol and were tumbled for an hour at 30 rpm. The tumbled samples were then centrifuged for 20 min at 3000 rpm before they were passed through a 0.45 micron filter. Sample pH was then adjusted to 7 via the addition of acidified MilliQ water. Surrogates and internal standards for all compounds were added prior to analysis, as described in Coggan et al. [17].

### 2.7. Bioconcentration Factors (BCFs) 

A reduction in PFAS concentration in the experiment could be due to compounds sticking to the experimental equipment such as the glass beakers, rather than the organisms. To account for this the concentration of PFAS in each experiment was measured both in the water and in the organisms at the end of the experiment via LC–MS/MS. This enabled us to confirm the actual concentration present, rather than just assuming the nominal experimental concentration was correct. We were also able to use these data to calculate the bioconcentration factor (BCF) of each compound.

BCF describes the readiness of chemicals to concentrate in organisms when the compounds are present in the environment. It is a required ecotoxicological parameter for chemical regulation and is generally taken to be the ratio of the concentration of a substance in a specific organism to the exposure concentration, at equilibrium in the media concerned. In this study, BCF was calculated via Equation (1):

BCF = C/Cw,(1)

where BCF = bioconcentration factor, C = the concentration of the test substance in the organism in µg/g and Cw = the concentration of the substance in water in µg/L.

### 2.8. Statistical Analysis—Survival

The effect of PFAS exposure on amphipod survival was assessed using Student’s *t*-test (two samples assuming equal variances). Each treatment was compared to controls and significance was set at *p* < 0.05 (Table S4).

### 2.9. Metabolite Data Analysis 

A combination of multivariate and univariate statistics were used for identification and selection of biomarkers using the total area of the chromatogram peak for each metabolite [18]. Each PFAS exposure was analysed separately. 

Values were normalized to the median value of each treatment to account for differences between samples [19]. Values were then transformed (log_natural_) to reduce variance between metabolite abundance to allow easier comparison between compounds of high and low concentrations. 

Multivariate statistical analysis was applied as a first step for interrogating the data to observe trends, grouping and/or outliers [18]. Given the organisms used in this study were originally wild caught and then cultivated, rather than a lab-based population, their genetic and metabolic variation was expected to be higher than lab grown organisms. It was therefore possible that natural biological variation might mask the effects of PFAS exposure, particularly at low concentration levels. Orthogonal Partial Least Squares—Discriminant Analysis (OPLS-DA) with appropriate model overview and Variable Importance for Projection (VIP) investigating the metabolites causing the shift between groups (supplied in Supplementary Materials—Figures S1–S17) is a common method for determining separation in metabolomic datasets due to treatments when natural biological variation outweighs treatment variation [20]. Therefore, OPLS-DA was carried out using online MetaboAnalyst Version 5.0 (https://www.metaboanalyst.ca/MetaboAnalyst/) (accessed on 15 January 2021) [21] for each experiment separately. 

A univariate Analysis Of VAriance (ANOVA) was also conducted to determine significant differences in metabolite levels between treatments (*p* < 0.05). Following this, Tukey’s post hoc test determined significance between treatments at each concentration. Metabolites were analysed using one-factor ANOVA with treatment as the main effect with R Version 3.0.3, R Foundation for Statistical Computing, Vienna, Austria [22]. Each *p*-value was adjusted for false discovery rate using the Benjamini–Hochberg (BH) method. The metabolic data were then graphed using box plots. Finally, pathway enrichment analysis was assessed using MetaboAnalyst, Version 5.0, (https://www.metaboanalyst.ca/MetaboAnalyst/) (accessed 15 January 2021) [21], to highlight potential pathways impacted by changes in metabolite abundance. 

## 3. Results and Discussion

### 3.1. Survival

**Figure 1:**
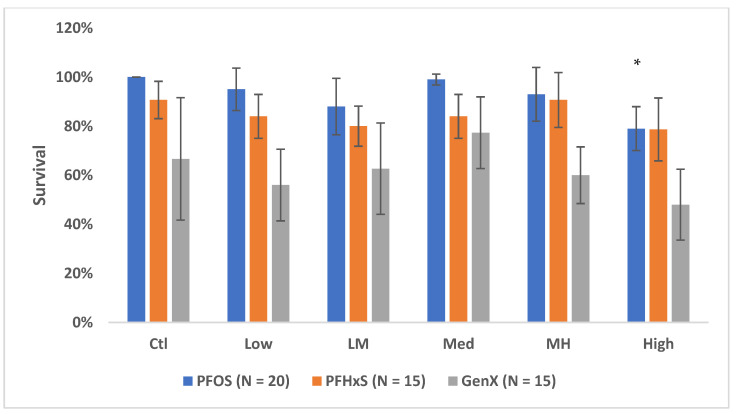
Mean (±SD) survival of amphipods *Austrochiltona subtenuis* following exposure to different concentrations of PFOS, PFHxS and GenX for 7 days. Two-tailed *t*-test between control and concentration revealed survival of amphipods were significantly reduced (*p* = 0.00) * at the highest PFOS concentration. Ctl = control, LM = low–medium, and MH = medium–high.

Survival varied across all treatments, with a decline in survival in the higher concentrations as shown in Figure 1. There was a decrease in total percent survival in high concentrations in all treatments, 21% (PFOS), 18% (GenX), and 12% (PFHxS), compared to the relevant controls. Only the high-dose group for PFOS had a significant reduction in survival compared to controls (*p* < 0.00) (Supplementary Materials, Table S4). 

The survival rates of the controls and the medium exposure were similar, with the controls a little lower than the medium in GenX. However, there is no statistical difference in the survival rates of each group. The variation is higher than ideal, but when dealing with a wild-caught population, some variation is expected; indeed, from our experience, it is not uncommon in this kind of work. Furthermore, the medium dose of GenX was far lower than that has been shown to cause an effect on survival, so we did not expect it to cause an effect. Crucially, this observation backs up the main point that traditional ecotoxicology tests are not sensitive enough to register the effects of PFAS at environmentally relevant exposures levels. In this case, no statistical difference in the survival rates of each group and so testing for survival would show no effects of PFAS exposure. It takes the metabolomics approach to determine the subtle differences of sublethal exposure levels. Moreover, the current paper is not looking at survival but at the effects of PFAS on metabolism at sublethal exposure levels. 

Additionally, at the end of the exposures, amphipods from the medium–high- and high-dose treatments for all compounds had visibly reduced biomass but this in itself was not enough to demonstrate an effect. This means that the Australian PFAS NEMP guideline for 80–99% protection of organisms at those concentrations is technically correct—if one is using survival as a metric. However, it is worth noting that the survival of the amphipods was affected following just 7 days of exposure and longer exposures may decrease the survival rates further. In comparison, the marine amphipod, *Melita plumulosa*, was described to have a PFOS 10-d LC_50_ of 3.3 mg/L and a reproduction EC_50_ of 2.2 mg/L [23]. These concentrations are significantly higher than those used in this study. Similarly, the common freshwater amphipod, *Hyalella azteca,* was reported to have a PFOS 42-d LC_50_ of 15 mg/L and an EC_50_ of 3.8 mg/L for reproduction [24]. This suggests amphipods can be considered tolerant of concentrations detected in the majority of PFAS-contaminated sites when using standard endpoints such as survival.

Boudreau et al. [25] stated that at the known environmental concentrations of PFAS, which range from ng/L to mg/L in ecosystems, there is seemingly no adverse risk of PFOS to freshwater ecosystems. This was after the authors had exposed a number of aquatic flora and invertebrates to short term 48 and 96 h PFOS and concluded that PFOS is highly toxic to freshwater systems at 100 mg/L [25]. These concentrations are far above the levels of PFAS in most systems. 

### 3.2. Metabolomics

A total of 27 metabolites were detected using GC–MS. These included amino acids (e.g., valine; proline), carbohydrates (e.g., glycerol), carboxylic acids (e.g., acetic acid; propanoic acid), lipids (e.g., cholesterol), fatty acids (e.g., palmitic acid) and glycerides (e.g., monostearin). Only metabolite features that were statistically significant between treatments were fully identified and used for the multi- and univariate analysis. 

### 3.3. PFOS Exposure

#### 3.3.1. Multivariate Data Analysis for PFOS Exposure

**Figure 2:**
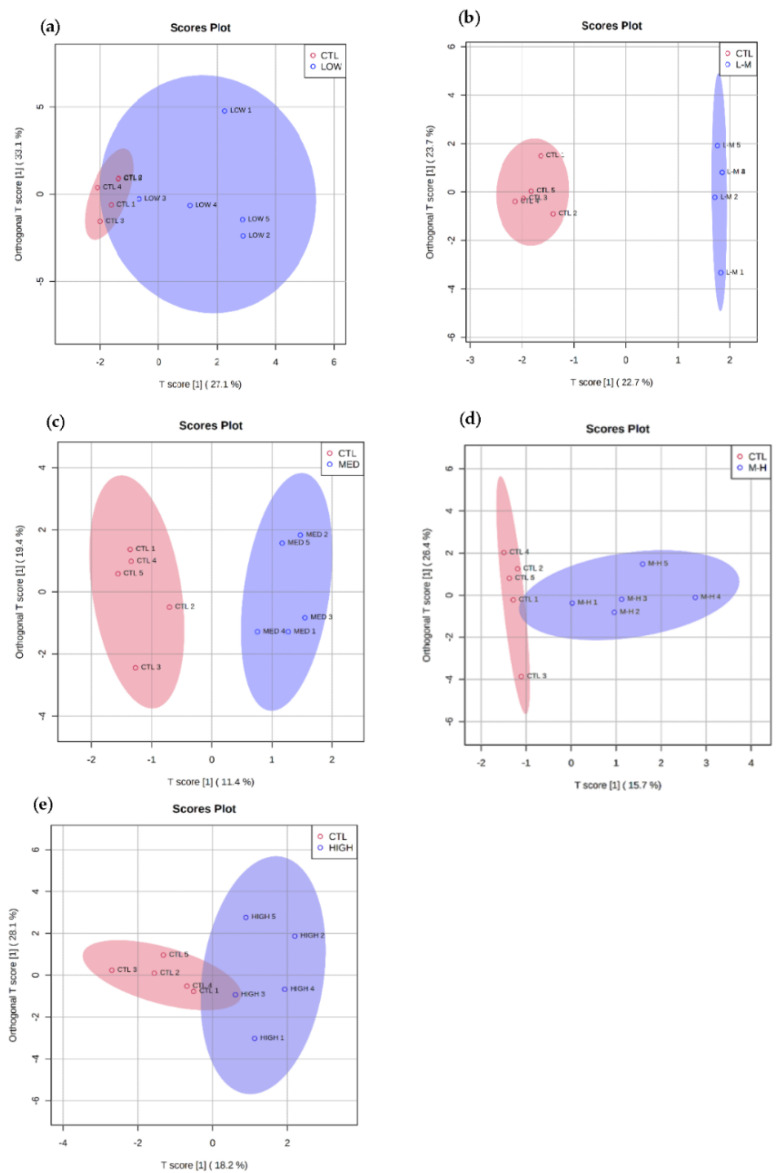
Orthogonal PLS-DA of polar metabolites from amphipods exposed to PFOS (blue) and control (pink) for 7 days at (**a**) low (0.04 µg/L), (**b**) low–medium (0.20 µg/L), (**c**) medium (1.00 µg/L), (**d**) medium–high (5.00 µg/L) and (**e**) high (25.00 µg/L) concentrations. Orthogonal PLS-DA model validation data can be found in Supplementary Materials (Figures S1–S5).

OPLS-PA provided a visual tool to assess the effects of PFOS on the amphipods at different concentrations. There was an overlap of the metabolomic profile responses in amphipods exposed to the low-dose and control groups (Figure 2a) but separation between controls and all other groups (Figure 2b–e).

The Variable Importance for Projection (VIP) indicated similar metabolites to the univariate analysis were responsible for driving the main separation (see Section 3.3.2 and Supplementary Materials, Figures S1–S5). Briefly, isopropyl propane, valine and propanoic acid were shown to be the cause of most of the separation in the lower concentration of PFOS, whereas palmitic acid was highlighted in the higher concentration. 

#### 3.3.2. Univariate Data Analysis for PFOS Exposure

Univariate analysis of significant metabolite features was used to further elucidate the overall effects of exposure to PFOS on amphipods. There were seven metabolites whose levels changed significantly (Table 2).

**Figure 3:**
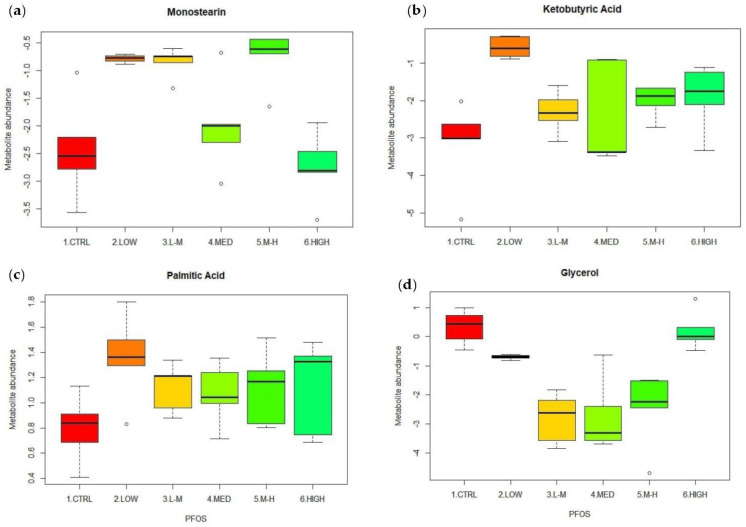
Top four significant metabolite features from amphipods when exposed to PFOS at low (0.04 µg/L), low–medium (0.20 µg/L), medium (1.00 µg/L), medium–high (5.00 µg/L) and high (25.00 µg/L) concentrations. (**a**) Monostearin; (**b**) ketobutyric acid; (**c**) palmitic acid; and (**d**) glycerol.

Box plots were used to display significantly responding metabolite abundance trends across the different concentrations of PFOS. There was an increase in monostearin total abundance compared to the controls in the low, low–medium and medium–high groups (Figure 3a), but a statistically significant response was only detected in the low–medium (*p* = 0.02) and medium–high groups (*p* = 0.01; Table 2). Ketobutyric acid had the highest, and statistically significant (*p* = 0.00; Table 2), increase in abundance in the low concentration group compared to the controls. There was also a trending increase in the abundance of this metabolite compared to the control in all groups (Figure 3b). Palmitic acid showed a statistically significant response (*p* = 0.00; Table 2) at the highest concentration (Figure 3c). Glycerol decreased in abundance in the low–medium group then increased in the M–H- and high-dose groups (Figure 3d). The abundance of propanoic acid, acetic acid and valine significantly changed in response to PFOS (Table 2), and these box plots are listed in Supplementary Materials (Figure S6). 

### 3.4. GenX Exposure

#### 3.4.1. Multivariate Data Analysis for GenX Exposure

**Figure 4:**
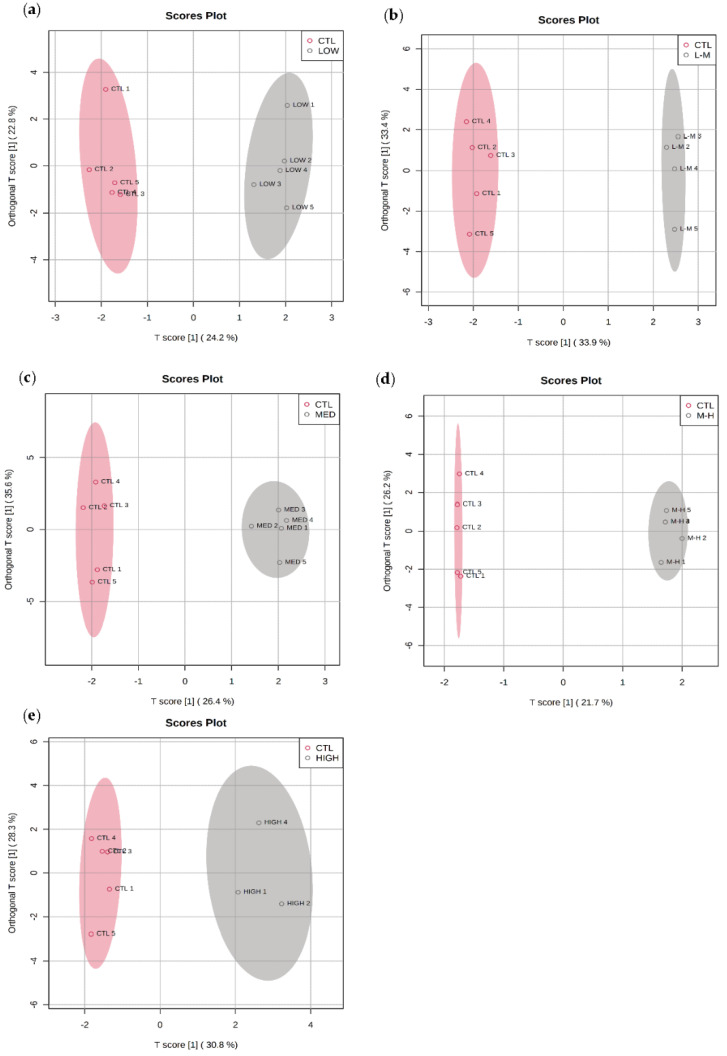
Orthogonal PLS-DA: separation of polar metabolites from amphipods exposed to GenX for 7 days at (**a**) low (0.03 µg/L), (**b**) low–medium (0.16 µg/L), (**c**) medium (0.80 µg/L), (**d**) medium–high (4.00 µg/L) and (**e**) high (20.00 µg/L) concentrations. Orthogonal PLS-DA Model overview can be found in Supplementary Materials (Figures S7–S11).

The amphipod metabolomic profiles in response to GenX had a distinct response compared to controls in each concentration (Figure 4a–e). There was a clear separation of groups from low to high concentrations.

Cholesterol, monostearin and glycerol were identified using the VIP for each concentration to be responsible for the separation between control and treatment in the OPLS-DA plots (Supplementary Materials, Figures S7–S11), these were later confirmed to be significantly changed via univariate analysis. 

#### 3.4.2. Univariate Data Analysis for GenX Exposure

An ANOVA was run for each metabolite following exposure to GenX to identify any significant responses (Table 3). 

**Figure 5:**
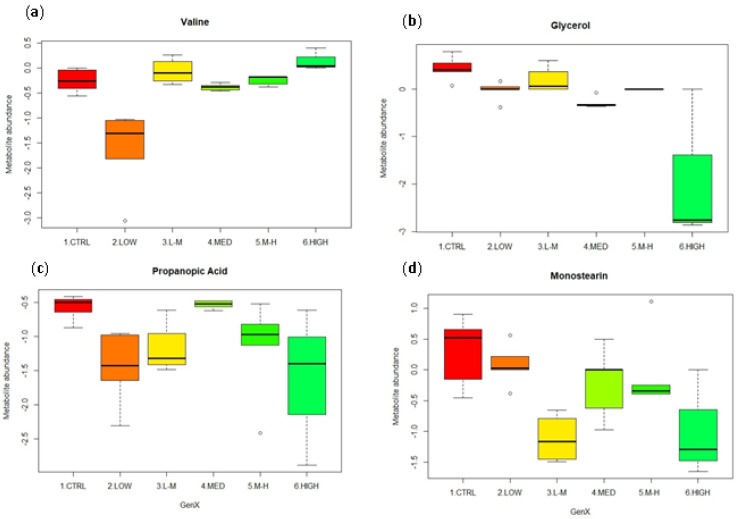
Significant metabolites from amphipods exposed to GenX at low (0.03 µg/L), low–medium (0.16 µg/L), medium (0.80 µg/L), medium–high (4.00 µg/L) and high (20.00 µg/L) concentrations. (**a**) Valine; (**b**) glycerol; (**c**) propanoic acid; (**d**) monostearin.

The metabolites showing the most change in each group were shown using box plots (Figure 5a–d).

Valine was significantly reduced in abundance only in the low concentration group compared to controls (Figure 5a) (*p* = 0.00; Table 3). Glycerol was significantly reduced in abundance following exposure to each concentration of GenX (Table 3) compared to the control (Figure 5b). Propanoic acid had similar trends to monostearin, with the abundance of both metabolites generally decreasing compared to controls (Figure 5c). The effect of GenX on monostearin started at the lower concentrations, decreased in the low–medium concentrations, increased at medium concentrations before further declining in the high treatments (Figure 5d). Overall, the abundance of these metabolites reduced in abundance compared to controls. Additionally, cholesterol and palmitic acid were found to significantly respond (Table 3), these box plots are located supplementary material (Figure S12a,b).

### 3.5. PFHxS Exposure

#### 3.5.1. Multivariate Data Analysis for PFHxS Exposure

**Figure 6:**
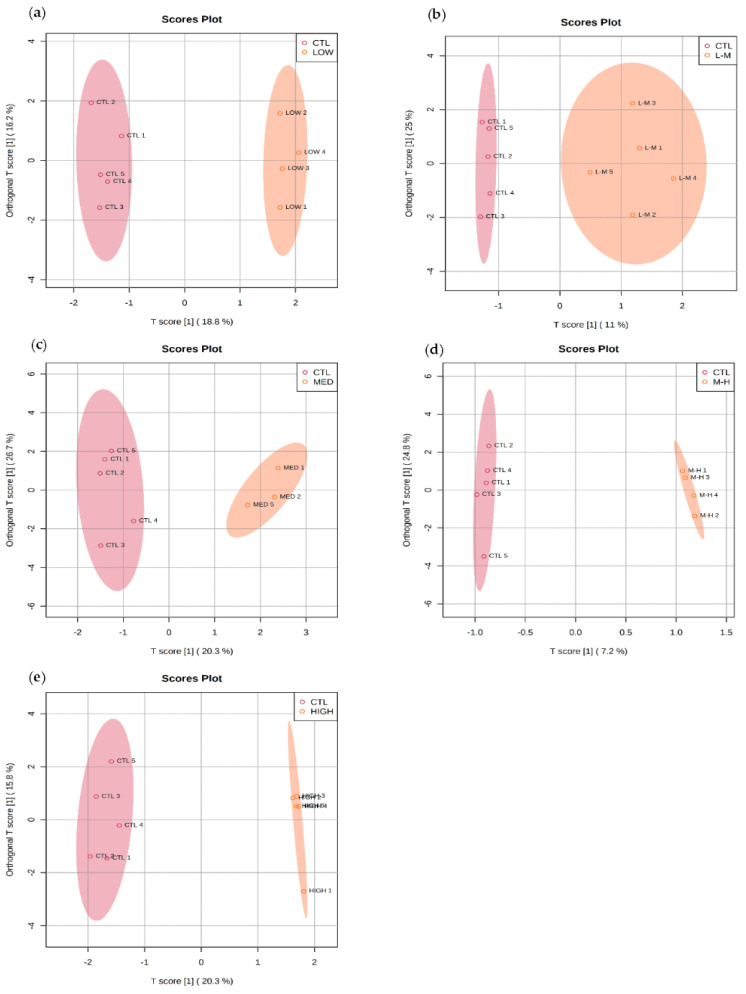
Orthogonal PLS-DA: separation of polar metabolites from amphipods exposed to PFHxS for 7 days at (**a**) low (0.03 µg/L), (**b**) low–medium (0.15 µg/L), (**c**) medium (0.76 µg/L), (**d**) medium–high (3.78 µg/L) and (**e**) high (18.92 µg/L) concentrations. Orthogonal PLS-DA model overview can be found in Supplementary Materials (Figures S13–S17).

OPLS-DA was used to visualise the effect of the different concentrations of PFHxS on the amphipod metabolomic profiles. Similar to the response seen for GenX, there was clear separation in metabolite abundance in amphipods exposed to low, L–M, medium, M–H and high PFHxS concentrations (Figure 6a–e). These results indicate that both the newer compounds (GenX and PFHxS) have a stronger effect on metabolism than PFOS even though the latter is currently thought to be of more concern.

The VIP for the OPLSDA highlighted similar metabolites that were identified for driving the separation in PFOS and GenX OPLSDA (Supplementary Materials, Figures S13–S17). Cholesterol, monostearin and propanoic acid were identified to be responsible for the separation between PFHxS and control treatments. These metabolites were then identified to be significant in the univariate analysis.

#### 3.5.2. Univariate Data Analysis for PFHxS Exposure

**Figure 7:**
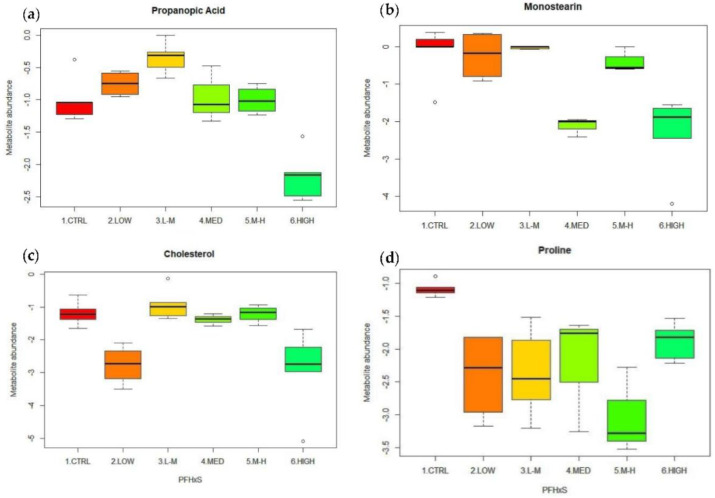
(**a**–**d**) Significant metabolites from amphipods exposed to PFHxS at low (0.03 µg/L), low–medium (0.15 µg/L), medium (0.76 µg/L), medium–high (3.78 µg/L) and high (18.92 µg/L) concentrations. (**a**) Propanoic acid; (**b**) monostearin; (**c**) cholesterol; (**d**) proline.

There were four metabolites that were identified as being statistically different in total abundance following exposure to PFHxS compared to controls (Table 4). Box plots were used to visualise the overall trend in the abundance of these four metabolites (Figure 7a–d).

Propanoic acid increased in abundance at the low concentration and spiked at the low–medium concentration (*p* = 0.04, Table 4) for PFHxS before dropping in total abundance at higher concentrations compared to the controls. This resulted in a significant reduction in abundance at a high concentration (*p* = 0.00, Table 4) (Figure 7a). There was a significant decline in abundance in the glyceride monostearin in the medium and high concentrations of PFHxS (Table 4), whereas there was little effect on monostearin abundance in the remaining concentrations, low, L–M and M–H (Figure 7b). PFHxS had a significant effect on the level of cholesterol, which was reduced in abundance at the low and high concentrations (*p* = 0.03, *p* = 0.01, respectively) (Table 4). However, cholesterol remained relatively consistent with control levels at the other concentrations (Figure 7c). Finally, the amino acid proline significantly decreased in abundance compared to the controls in three of the five concentrations of PFHxS (Table 4 and Figure 7d).

### 3.6. Overall PFAS Effect on Metabolites

Using metabolomics, we can begin to monitor, and rapidly screen, the small biological molecules in an organism at a given point in time and use these to help to elucidate the biological effects of PFAS [4,26]. The univariate analysis for each PFAS exposure highlighted significantly responding metabolites at different concentrations. There are several metabolites that were found to have a significant response in two and three of the PFAS exposures. For example, monostearin, palmitic acid and propanoic acid had a similar response in each PFAS exposure. It is however worth noting that the responses of the metabolites did not line up perfectly for each compound and PFAS concentration. For example, an increase in propanoic acid was detected in the low PFOS exposure, in the L–M group for PFHxS and the medium concentration of GenX. PFOS and GenX had similar effects on palmitic acid at each concentration, slightly dropping in abundance in the first three concentrations but increasing at high concentrations. This indicates that there may be different biological responses to low-dose PFAS exposure compared to high-dose exposure. The metabolites that change are all involved with fatty acid metabolism, potentially indicating an identifiable biological effect of exposure to fluorinated compounds. The result of fatty acid metabolism being impacted by PFAS exposure is consistent with the recent review investigating common biochemical pathways in response to PFAS [5]. This review highlighted those effects on fatty acid metabolism were seen in a range of organisms following PFAS exposure whether assessed by transcriptomics, lipidomics, or metabolomics [5].

### 3.7. Metabolomic Pathways

The biochemical pathways potentially disrupted due to exposure to the PFAS treatments were amino sugar metabolism; aspartate metabolism; pyruvate metabolism (e.g., acetic acid *p* = 0.04); steroid biosynthesis; steroidogenesis; bile acid biosynthesis (e.g., cholesterol, *p* = 0.00; palmitic acid, *p* = 0.00); glycerolipid metabolism (e.g., glycerol, *p* = 0.00; palmitic acid, *p* = 0.00); galactose metabolism (e.g., glycerol, *p* = 0.00); fatty acid metabolism; fatty acid elongation (e.g., palmitic acid, *p* = 0.00, monostearin, *p* = 0.00); propanoate metabolism (e.g., ketobutyric acid, *p* = 0.00; propanoic acid, *p* = 0.03); valine, leucine and isoleucine degradation (e.g., valine, *p* = 0.05); arginine and proline metabolism (e.g., Proline, *p* = 0.00). 

**Figure 8:**
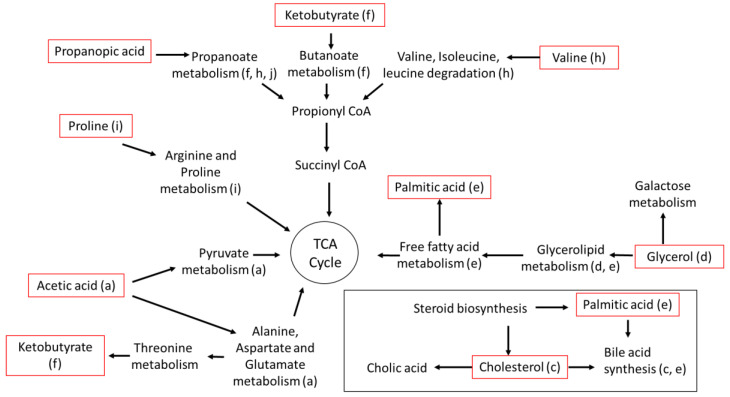
Metabolic pathways affected by PFAS exposure following a 7-day exposure. Red outline indicates the metabolites that changed significantly in abundance (listed in Table 2, Table 3 and Table 4).

Disruption in the pathways outlined in Figure 8 can impact the organism’s energy balance but this would likely be a general toxicant response and not a response to PFAS specifically. Fatty acid metabolism was affected, affecting the maintenance and development of the cuticle, leading to a delay in molts, growth, and development [27,28]. There is a possibility that reduced growth and size could lead to a decline in total population fitness over time, but more work would be needed to assess this. 

There was a change in the metabolite profile of each treatment at every concentration of PFAS compared to controls. This response was dose and contaminant dependent. Therefore, a linear response to PFAS cannot be assumed when organisms are exposed to increasing concentrations of these compounds. Studies investigating the effect of PFAS exposure on humans have made similar observations, where PFAS had a non-monotonic response in relation to kidney disease, cognitive function, diabetes, and fertility [29,30,31,32]. The results shown here challenge the common perception that increasing concentration equals increasing toxicity and accumulation of PFAS in a linear fashion. Instead, the data suggest metabolic responses can vary depending on the level of exposure.

Exposure to PFAS has been shown to increase fatty acid and energy metabolism in a range of aquatic organisms—for example, the water flea *Daphnia magna* [33] and the eastern school prawn *Metapenaeus macleayi* [34]. A study of turtles from PFAS-impacted waterways in Queensland, Australia, found an inflammation response and re-routing of central carbon metabolites [4]. With many studies being published, similar metabolites/pathways are emerging across organisms. In a recent review, Beale et al. [5], listed the common biochemical pathways affected following PFAS exposure that were also detected in this study. These alterations could result in metabolic deficiencies or perturbations that might lead to a decline in physical condition, fertility, and survival.

### 3.8. PFAS Water Concentration 

A sample from the high-dose group from each treatment was measured via LC–MS (Table 5). A large reduction in PFOS concentration compared to the nominal concentration was observed, with a slightly smaller reduction for PFHxS. In contrast, there was little reduction in GenX levels in the water between the start and end of the experiment. This could be due to GenX binding with the equipment used, such as the beakers or substrate, rather then accumulating in the amphipods but steps were taken to reduce this during sample preparation and analysis, as per Coggan et al. [17].

### 3.9. Bioconcentration Factors

PFAS concentrations were below the limit of detection in the amphipods measured from low, low–med and medium concentrations. The BCFs at a high concentration of each compound are shown in Table 6. Only PFOS was found to bioaccumulate—GenX was quickly eliminated from the amphipods, and PFHxS was eliminated but at a slower rate than GenX. Exposure to PFHxS and GenX still induced metabolic changes even though BCFs were low. Only four of the eight metabolites detected to significantly change in response to PFAS did so in correlation with bioaccumulation. This would seem to indicate that bioaccumulation is not necessarily needed for a metabolic response to occur in an organism due to PFAS exposure.

PFOS has been reported to have high bioaccumulation potential in invertebrates, fish, reptiles, birds and mammals [4,13,35,36,37,38]. This was also found in the current study, where the amphipods had accumulated a greater amount of PFOS compared to GenX and PFHxS following the 7-day exposure. It is however important to note that studies on the bioaccumulation of PFAS are variable and not necessarily reported in a standardised fashion (e.g., dry weight versus wet weight), which limits their effectiveness in some cases. McCarthy et al. [39] also highlight the limited data that have been collected for fish, benthic organisms, and terrestrial biota (including reptiles and amphibians) and state that toxicology testing across all classes of organisms has not been consistent [39]. Additionally, Taylor et al. [34] noted that differences among individuals, species and environments can lead to a highly variable bioaccumulation of PFAS in aquatic systems. Despite this, it is still generally acknowledged that toxicity must come with bioaccumulation [40]. For example, Karnjanapiboonwong et al. [41] investigated the response of earthworms (*Eisenia fetida*) to a range of PFAS. They found that toxicity and accumulation of PFAS increased with increasing chain length, and similar results were reported by Taylor et al. [34]. 

Chain length is thought to also affect how and which PFAS are metabolised and excreted from organisms [33,37,42,43,44,45,46]. For example, GenX (C6) did not appear to have bioaccumulation potential when exposed to small fish, and it was hypothesised to be eliminated after ingestion in comparison to PFOS (C8) [13]. This result from Hassell et al. [13] is consistent with the GenX and PFOS exposure seen in this study using amphipods. PFOS accumulated in the fish and the amphipods, whereas GenX had the lowest BCF in the amphipods, either due to lack of uptake or rapid elimination. GenX is a relatively new/emerging PFAS, and little research has been carried out on its bioaccumulation potential in organisms. This should be addressed in future studies given these chemicals are expected to accumulate in ecosystems over a period of time [11,47].

PFHxS is also a C6 fluoro-carbon compound and, as such, it is assumed to have a similarly low bioaccumulation potential. In this study, the BCF of PFHxS was in between that of PFOS and GenX, in agreement with studies claiming BCF increases with increasing chain length [48]. However, chain length may not be the only factor determining the BCF of these compounds. It is possible that the shape and overall width of the molecule may also need to be considered.

## 4. Conclusions

This is the first study investigating the effects of PFAS using metabolomics on the freshwater amphipod *Austrochiltonia. subtenuis*. The work shows that metabolomics can add value to ecotoxicology studies on PFAS. Metabolic effects were observed at lower concentrations of PFAS than were seen with more traditional ecotoxicological endpoints such as survival or bioaccumulation, indicating that our understanding of the effects of these compounds is not complete. Metabolomic pathways detected to be affected by PFAS exposure have been shown to respond in other biota exposed to PFAS. These pathways include fatty acid, arginine, proline, glycolipid, galactose, and aspartate metabolism. We recommend additional research on the metabolome of invertebrates to achieve a detailed understanding of the entire consequence of these changes. Here, we recommend further investigations using omic techniques to better understand the sublethal effects of PFAS on organisms at environmentally relevant concentrations—specifically investigating the effect on a variety of organisms and investigating the bioaccumulation of PFAS detected in ecosystems as well as investigating whether temperature, salinity and other parameters impact the metabolomic responses seen to be affected by PFAS and bioaccumulation. With a larger variety of studies, confidence in the sublethal effects of PFAS will continue to emerge. This study increases our overall understanding of PFAS toxicity at environmentally relevant concentrations. With further investigations, metabolite responses could potentially help PFAS-monitoring remediation and efforts to be better targeted.

## Figures and Tables

**Table 1:** Nominal concentrations (µg/L) of PFOS, GenX and PFHxS used in three separate *Austrochiltonia subtenuis* 7-day exposures using analytical-grade PFAS standards.

Treatment	Low	Low–Medium	Medium	Medium–High	High
Control	0	0	0	0	0
PFOS	0.04	0.20	1.00	5.00	25.00
GenX	0.03	0.16	0.80	4.00	20.00
PFHxS	0.03	0.15	0.76	3.78	18.92

**Table 2:** Significantly changed metabolites following exposure to PFOS identified from a one-way ANOVA. Values in bold represent statistical significance following Tukey’s post hoc test. All values listed are BH-adjusted *p* values (*p* < 0.05), using a 95% family-wise confidence level. Degrees of freedom = 24/5.

Metabolite	Mean Square Residual	Comparison between Treatment and Control				
		LOW-CTRL	L–M–CTRL	MED–CTRL	M–H–CTRL	HIGH–CTRL
Monostearin	3 × 10^11^	0.09	0.02	1.00	0.01	0.85
Ketobutyric acid	2 × 10^11^	0.00	1.00	0.70	0.96	0.41
Palmitic acid	3 × 10^11^	0.17	1.00	1.00	0.08	0.00
Glycerol	1 × 10^13^	0.02	0.01	0.01	0.01	0.93
Propanoic acid	2 × 10^11^	0.74	0.95	0.99	0.52	0.50
Acetic acid	2 × 10^14^	1.00	0.11	1.00	0.99	0.94
Valine	1 × 10^13^	0.20	0.06	0.72	0.12	0.08

**Table 3:** Significantly changed metabolites in amphipods following exposure to GenX. Values in bold represent statistical significance following Tukey’s post hoc test. All values listed are BH-adjusted *p* values (*p* < 0.05) using a 95% family-wise confidence level. Degrees of freedom = 21/5.

Metabolite	Mean Square Residual	Comparison between Treatment and Control				
		LOW-CTRL	L–M–CTRL	MED–CTRL	M–H–CTRL	HIGH–CTRL
Valine	9 × 10^10^	0.00	1.00	0.75	0.75	0.56
Glycerol	9 × 10^10^	0.01	0.04	0.00	0.01	0.00
Monostearin	9 × 10^10^	0.44	0.74	0.03	0.87	0.70
Propanopic acid	9 × 10^10^	0.01	0.02	1.00	0.04	0.02
Cholesterol	9 × 10^10^	0.27	0.44	0.00	0.23	0.10
Palmitic acid	9 × 10^10^	0.65	0.48	0.19	0.12	0.02

**Table 4:** Significantly changed metabolites identified from a one-way ANOVA following exposure to PFHxS compared to controls. Values in bold represent statistical significance following Tukey’s post hoc test. All values listed are BH-adjusted *p* values (*p* < 0.05) using a 95% family-wise confidence level. Degrees of freedom = 20/5.

Metabolite	Mean Square Residual	Comparison between Treatment and Control				
		LOW-CTRL	L–M-CTRL	MED-CTRL	M–H-CTRL	HIGH-CTRL
Propanoic acid	0.10	0.86	0.04	1.00	1.00	0.00
Monostearin	0.43	1.00	1.00	0.01	0.99	0.00
Cholesterol	0.48	0.03	0.99	1.00	1.00	0.01
Proline	0.31	0.02	0.02	0.10	0.00	0.25

**Table 5:** Nominal and Measured PFAS concentrations (µg/L)in treatments (PFOS, GenX and PFHxS). Control water < 0.01 µg/L of PFOS and PFHxS and < 0.1 µg/L of GenX (limit of detection).

Treatment	Day 0 (Nominal)	Day 7 (Measured)
PFOS	25.00	0.11
GenX	20.00	19.71
PFHxS	18.92	12.3

**Table 6:** Bioconcentration factors for each treatment of PFAS at a high concentration (µg/L).

Treatment	High R1	High R2	High R3	High R4	High R5
PFOS	18.18	27.27	190.91	118.18	9.09
GenX	0.01	0.01	0.01	0.01	0.01
PFHxS	0.05	0.07	0.04	0.08	0.03

## Data Availability

The data presented in this study are available in article and Supplementary Materials.

## Supplementary Materials

The following supporting information can be downloaded at: https://www.mdpi.com/article/10.3390/metabo12111135/s1, Table S1: Water quality for amphipod PFAS exposure (PFOS, GenX and PFHxS). Table S2: Total survival of amphipods following PFAS exposure (PFOS, GenX and PFHxS). Table S3: Retention time and *m*/*z* of significant metabolites detected. Table S4: Survival *t*-test (two samples assuming equal variance). Figures S1–S5: Orthogonal Partial Lease Squares—Discriminant Analysis (OPLS-DA)—amphipod separation PFOS model overview and VIP. Figure S6a–c: Significant metabolite features from amphipods when exposed to PFOS at low (0.04 µg/L), low–medium (0.20 µg/L), medium (1.00 µg/L), medium–high (5.00 µg/L) and high (25.00 µg/L) concentrations. (a) Propanoic acid; b) acetic acid c) valine. Figure S7–S11: Orthogonal Partial Lease Squares—Discriminant Analysis (OPLS-DA)—amphipod separation to GenX model overview and VIP. Figure S12a,b: Significant metabolites from amphipods exposed to GenX at low (0.03 µg/L), low–medium (0.16 µg/L), medium (0.80 µg/L), medium–high (4.00 µg/L) and high (20.00 µg/L) concentrations. (a) Cholesterol and (b) palmitic acid. Figures S13–S17: Orthogonal Partial Lease Squares—Discriminant Analysis (OPLS-DA)—amphipod separation following low concentration to PFHxS model overview and VIP.
