# Supplementary material for: Exposure to Environmentally Relevant Levels of PFAS Causes Metabolic Changes in the Freshwater Amphipod Austrochiltonia subtenuis
Source: Metabolites. 2022 Nov 18;12(11):1135. doi: 10.3390/metabo12111135 (PMC9698423; doi:10.3390/metabo12111135)
Supplement: Supplementary file 1 [file metabolites-12-01135-s001.zip › metabolites-2022916-supplementary.pdf]

Supplementary material

Table S1. Water quality for amphipod PFAS exposure (PFOS, GenX and PFHxS).

	Treatment	DO (%)	pH	Cond (uS/cm)	Temp (ºC)	NH+ (ppm)
PFOS	*Day 0*	96.6	8.25	1206	22	0
	Day 7					
	Control	106.6	8.08	1244	20.3	0
	Low	108.4	8.26	1255	20.8	0
	Low -Medium	109.6	8.28	1245	21.1	0
	Medium	108.6	8.13	1228	20.3	0
	Medium – High	110.4	8.3	1230	21.2	0
	High	99	8.12	1230	20.9	0
GenX	*Day 7*					
	Control	87.2	7.88	1401	20.1	0
	Low	91	8.13	1385	21.7	0
	Low -Medium	90.2	8.16	1389	20.6	0
	Medium	91.1	8.33	1374	19.8	0
	Medium – High	87.6	8.14	1373	19.7	0
	High	83.4	8.28	1460	20.9	0
PFHxS	*Day 7*					
	Control	91.5	7.92	1290	20	0
	Low	87.7	8.12	1280	21.2	0
	Low -Medium	86.4	8.11	1277	20.9	0
	Medium	88.4	8.16	1281	20.4	0
	Medium – High	81.8	8.19	1277	21.2	0
	High	83.2	8.03	1263	20.9	0

Table S2. Total Survival of Amphipods following PFAS exposure (PFOS, GenX and PFHxS).

Treatment	Replicate	PFOS (n=20)	GenX (n=20)	PFHxS (n=15)
**Control**	**1**	20	15	15
	**2**	20	7	12
	**3**	20	8	14
	**4**	20	13	14
	**5**	20	7	13
**Low**	**1**	20	6	12
	**2**	20	8	11

	**3**	19	12	14
	**4**	16	8	12
	**5**	20	8	14
**Low – Medium**	**1**	17	10	10
	**2**	20	6	12
	**3**	20	12	12
	**4**	16	12	13
	**5**	15	7	13
**Medium**	**1**	19	8	14
	**2**	20	13	12
	**3**	20	13	12
	**4**	20	13	14
	**5**	20	11	11
**Medium -High**	**1**	18	8	15
	**2**	20	12	11
	**3**	20	8	14
	**4**	20	9	15
	**5**	15	8	13
**High**	**1**	17	8	10
	**2**	15	5	15
	**3**	13	10	11
	**4**	17	5	12
	**5**	17	8	11

Table S3. Retention time and M/Z of significant metabolites.

**Metabolite**	**Retention Time (mins)**	**Mass to charge (M/z)**
Monostearin	14.30	43.96
Ketobutyric acid	6.68	74.98
Palmitic Acid	11.65	313.24
Glycerol	7.92	74.97
Propanoic acid	6.62	74.98
Acetic acid	7.17	74.97
Valine	6.88	74.98
Cholesterol	17.63	43.98
Proline	9.32	74.99

Table S4. Survival t-Test (Two-sample assuming equal variance). Degrees of freedom = 8.

**Treatment**		**Control – Low**	**Control – Low-Medium**	**Control - Medium**	**Control – Medium -High**	**Control – High**
**PFOS**	P(T<=t) two-tail	0.23	0.05	0.35	0.19	0.00
**GenX**		0.43	0.78	0.43	0.60	0.19
**PFHxS**		0.24	0.06	0.24	1.00	0.11

PFOS OPLSDA Model Overview and VIP

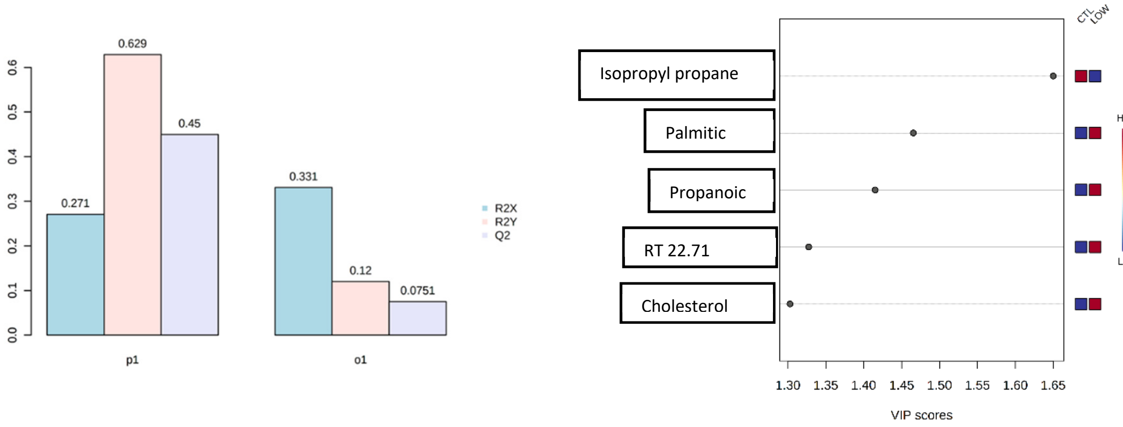

Figure S1. Low PFOS concentration Orthogonal Partial Least Squares - Discriminant Analysis (OPLS-DA) model overview and Variable Importance for Projection (VIP).

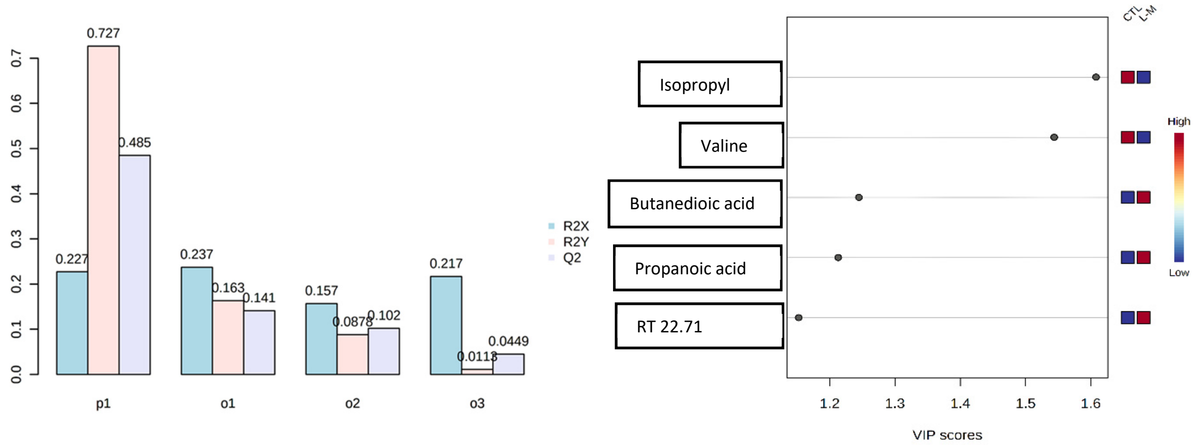

Figure S2. Low- medium PFOS concentration Orthogonal Partial Least Squares - Discriminant Analysis (OPLS-DA) model overview and Variable Importance for Projection (VIP).

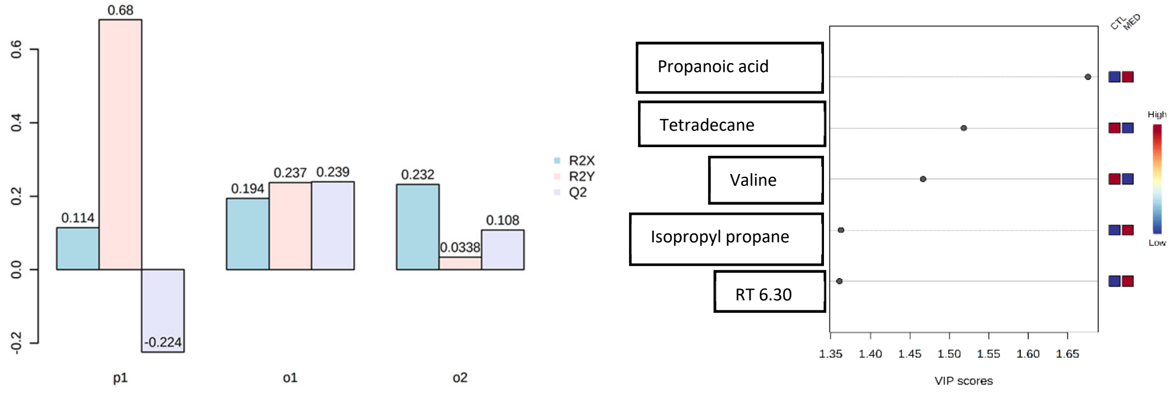

Figure S3. Medium PFOS concentration Orthogonal Partial Least Squares - Discriminant Analysis (OPLS-DA) model overview and Variable Importance for Projection (VIP).

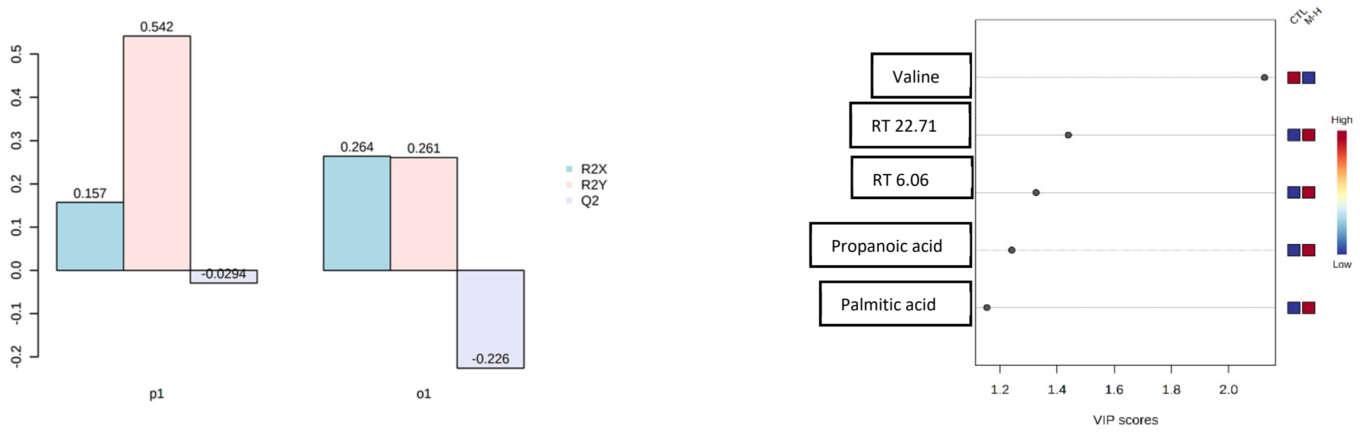

Figure S4. Medium – high PFOS concentration Orthogonal Partial Least Squares - Discriminant Analysis (OPLS-DA) model overview and Variable Importance for Projection (VIP).

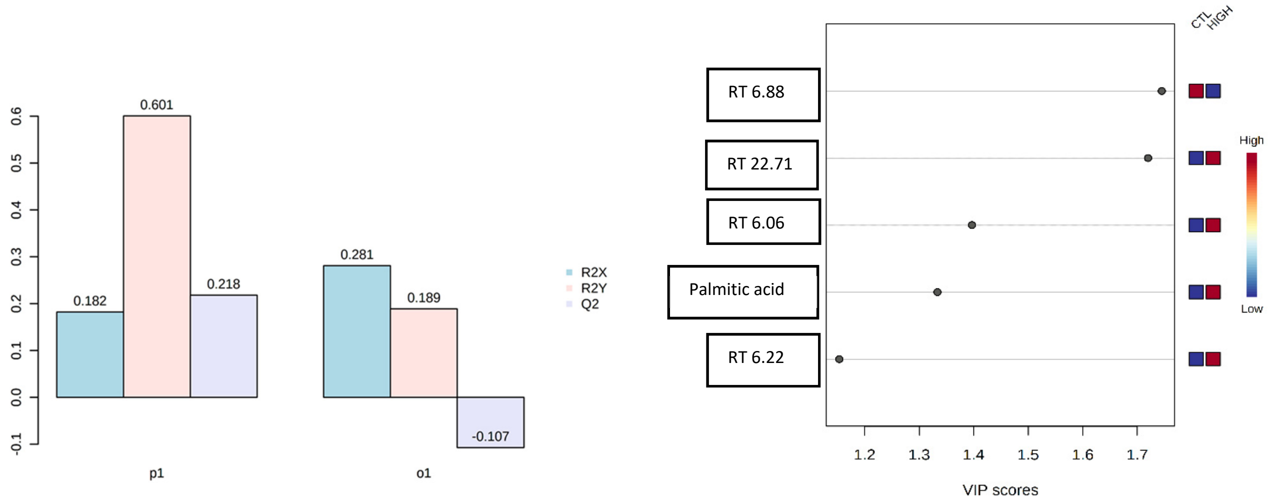

Figure S5. Medium – high PFOS concentration Orthogonal Partial Least Squares - Discriminant Analysis (OPLS-DA) model overview and Variable Importance for Projection (VIP).

Box plots PFOS

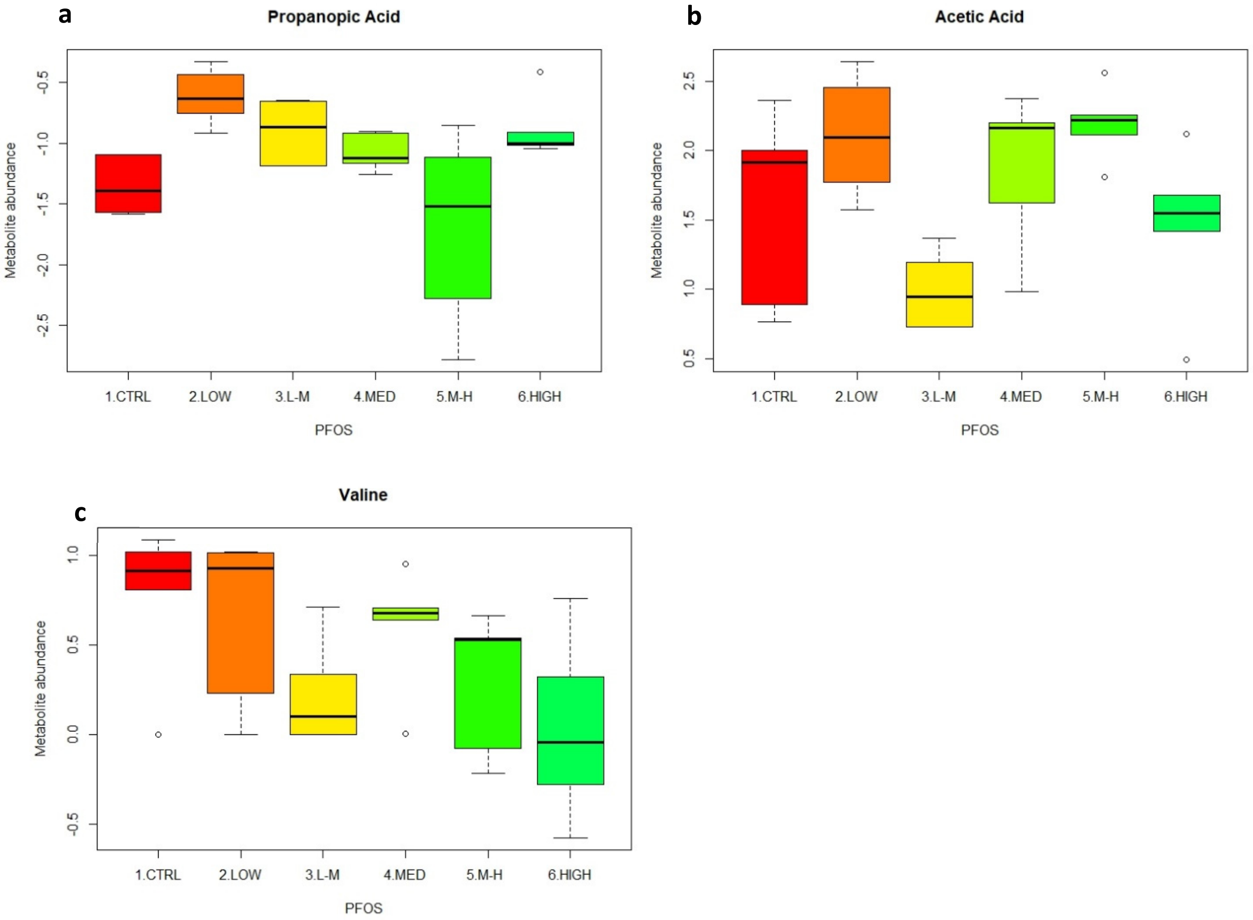

Figure S6. a-c). Significant metabolite features from amphipods exposed to PFOS - Low (0.04 ug/L), Low-Medium (0.20 ug/L); Medium (1.00 ug/L); Medium- High (5.00 ug/L) and High (25.00 ug/L). a) Propanoic Acid; b) Acetic Acid c) Valine.

GenX OPLS-DA Model Overview

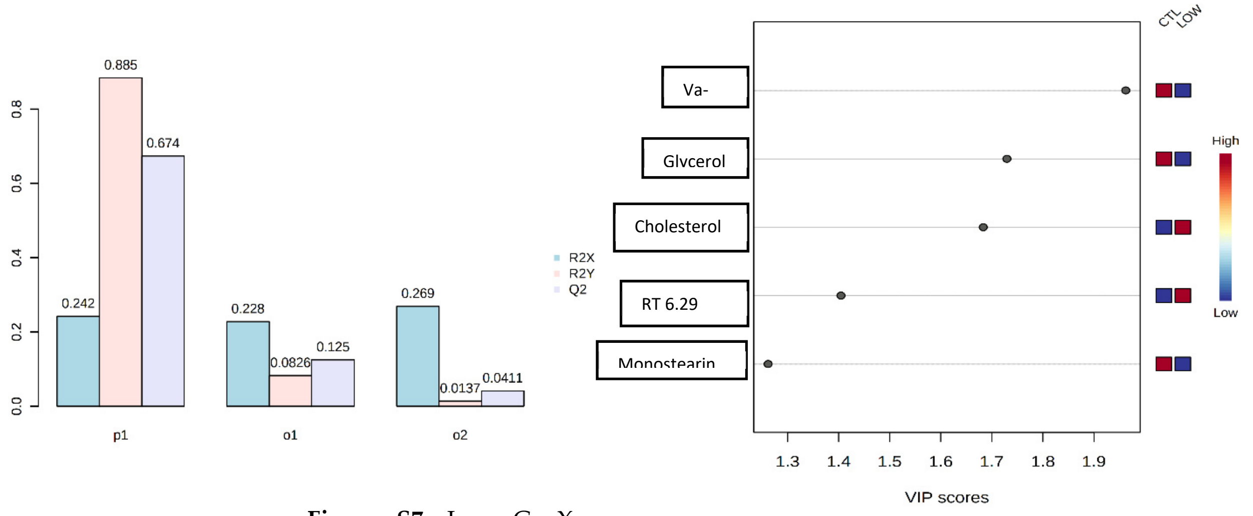

Figure S7. Low GenX concentration Orthogonal Partial Least Squares - Discriminant Analysis (OPLS-DA) model overview and Variable Importance for Projection (VIP).

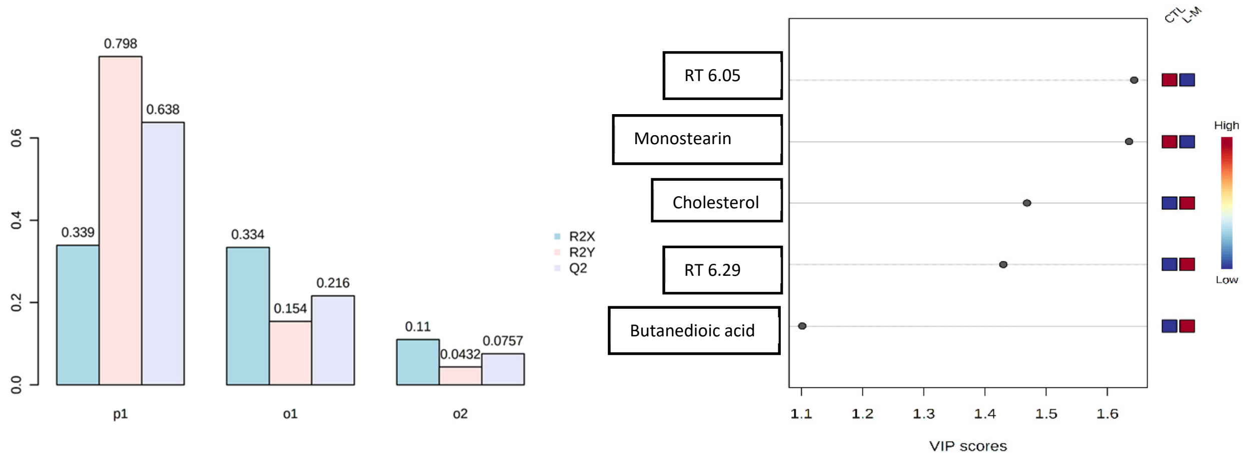

Figure S8. Low – medium GenX concentration Orthogonal Partial Least Squares - Discriminant Analysis (OPLS-DA) model overview and Variable Importance for Projection (VIP).

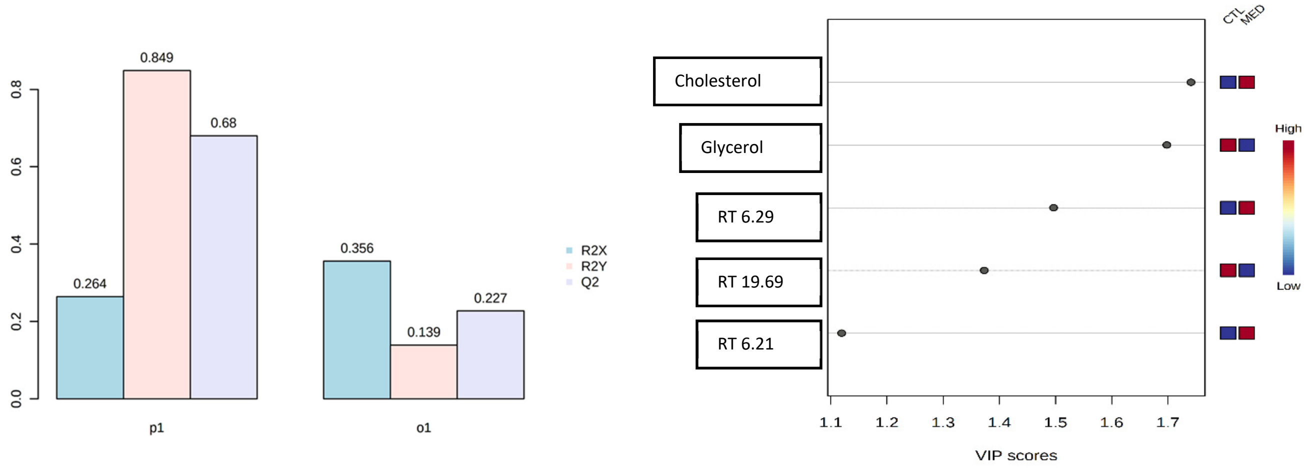

Figure S9. Medium GenX concentration Orthogonal Partial Least Squares - Discriminant Analysis (OPLS-DA) model overview and Variable Importance for Projection (VIP).

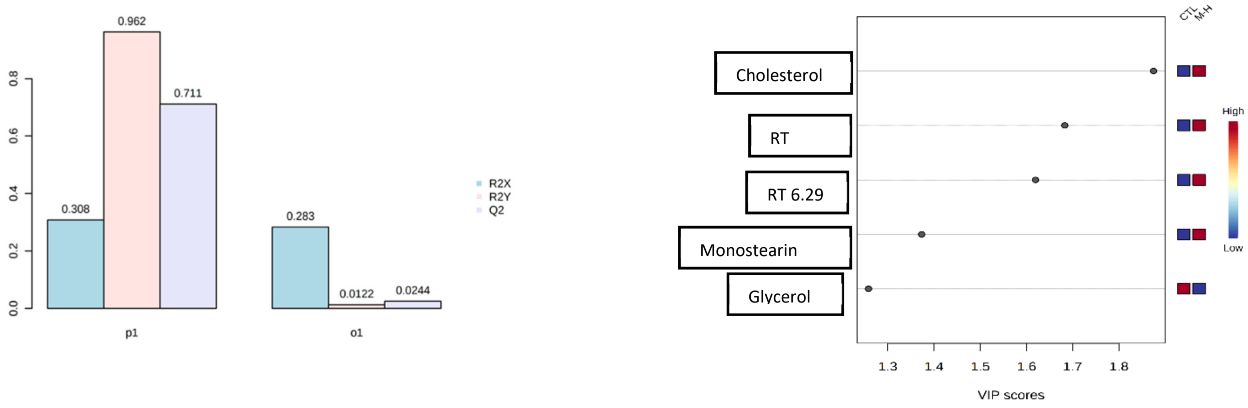

Figure S10. Medium- high GenX concentration Orthogonal Partial Least Squares - Discriminant Analysis (OPLS-DA) model overview and Variable Importance for Projection (VIP).

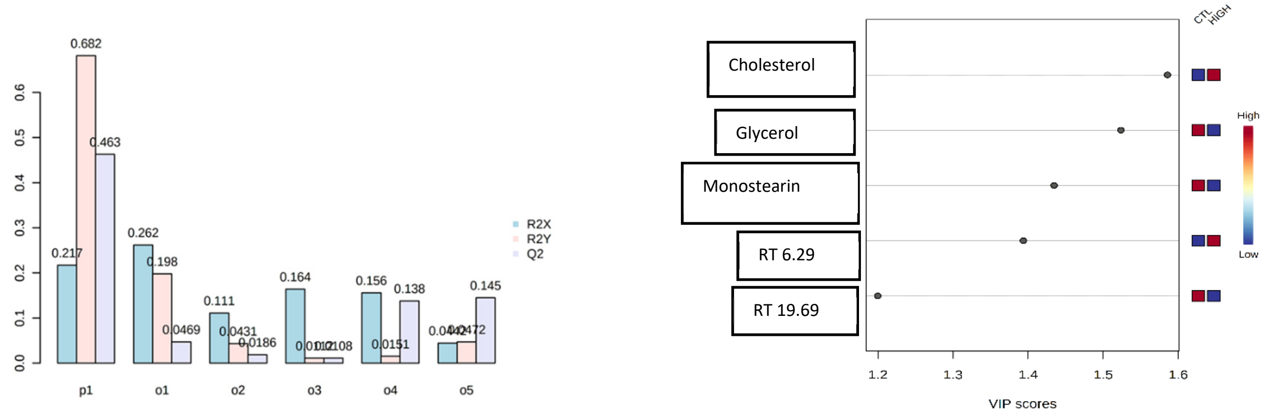

Figure S11. High GenX concentration Orthogonal Partial Least Squares - Discriminant Analysis (OPLS-DA) model overview and Variable Importance for Projection (VIP).

Box plots GenX

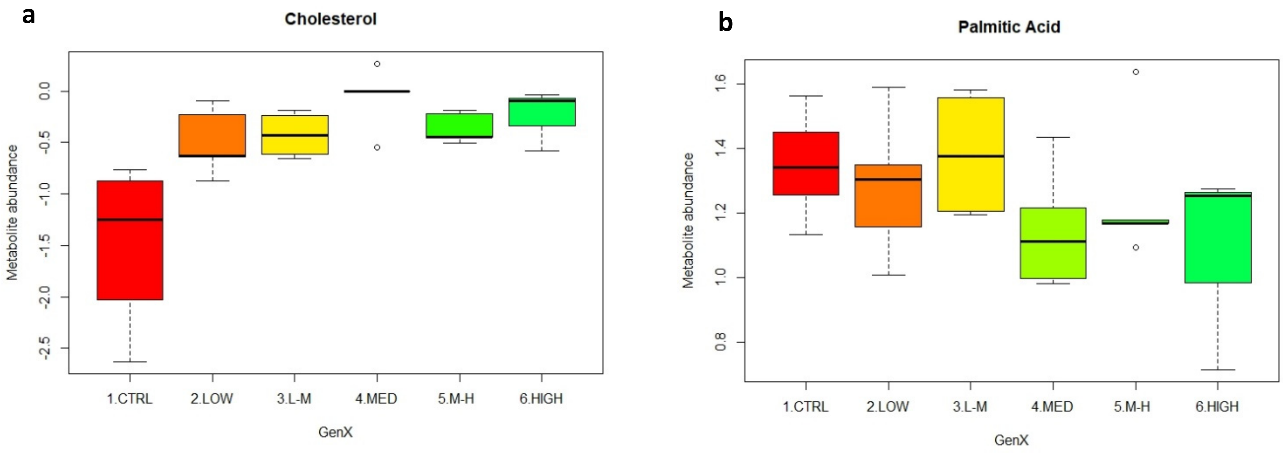

Figure S12. a-b) Significant metabolites from amphipods exposed to GenX Low (0.03 ug/L); Low-Medium (0.16 ug/L); Medium (0.80 ug/L); Medium- High (4.00 ug/L) and High (20.00 ug/L). a) Cholesterol and b) Palmitic acid.

PFHxS OPLS-DA Model Overview

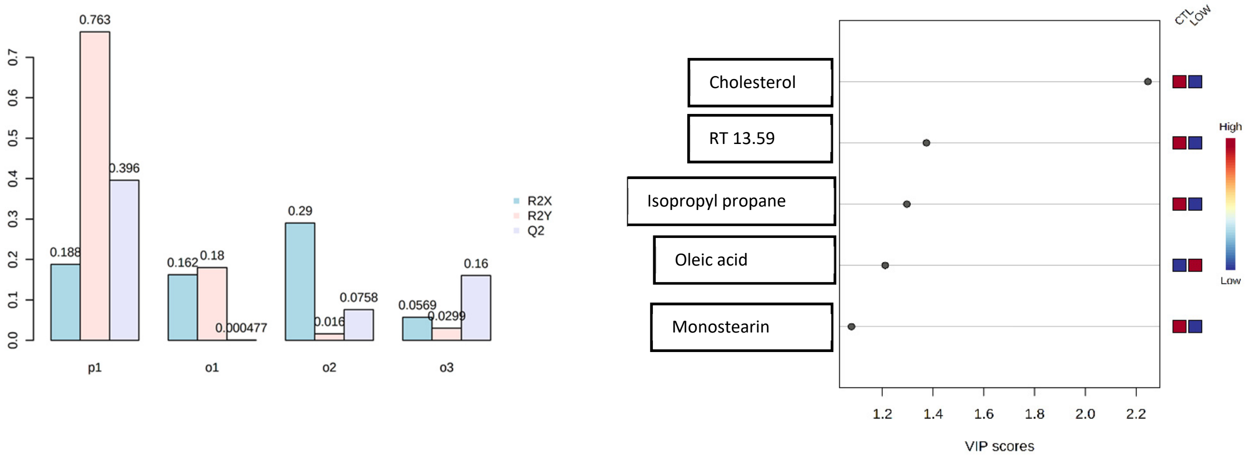

Figure S13. Low PFHxS concentration Orthogonal Partial Least Squares - Discriminant Analysis (OPLS-DA) model overview and Variable Importance for Projection (VIP).

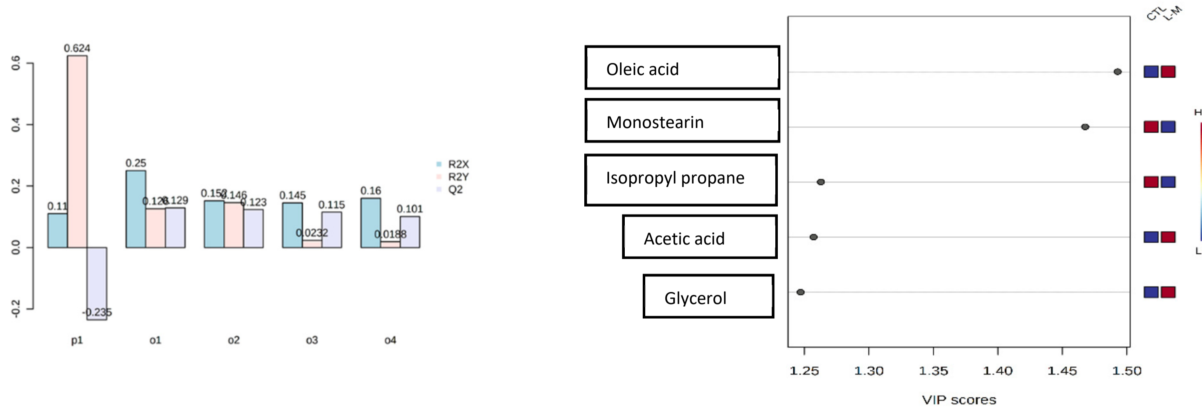

Figure S14. Low – medium PFHxS concentration Orthogonal Partial Least Squares - Discriminant Analysis (OPLS-DA) model overview and Variable Importance for Projection (VIP).

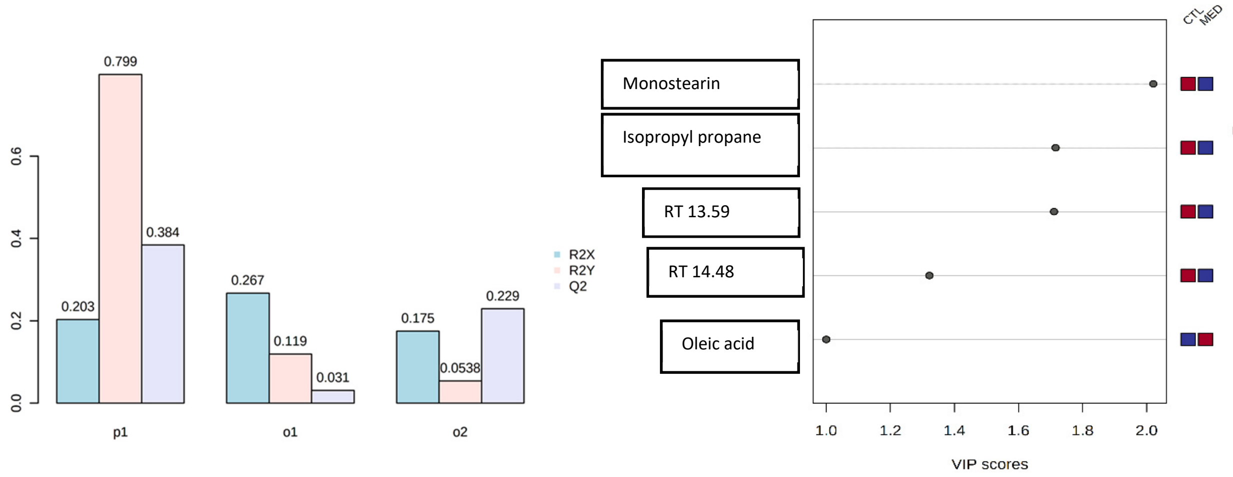

Figure S15. Medium PFHxS concentration Orthogonal Partial Least Squares - Discriminant Analysis (OPLS-DA) model overview and Variable Importance for Projection (VIP).

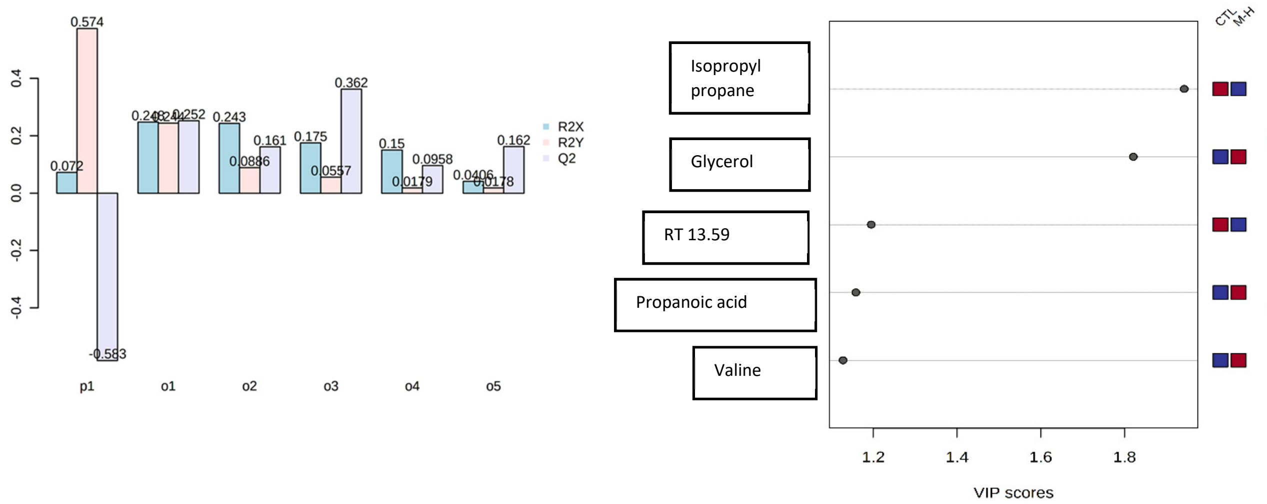

Figure S16. Medium- high PFHxS concentration Orthogonal Partial Least Squares - Discriminant Analysis (OPLS-DA) model overview and Variable Importance for Projection (VIP).

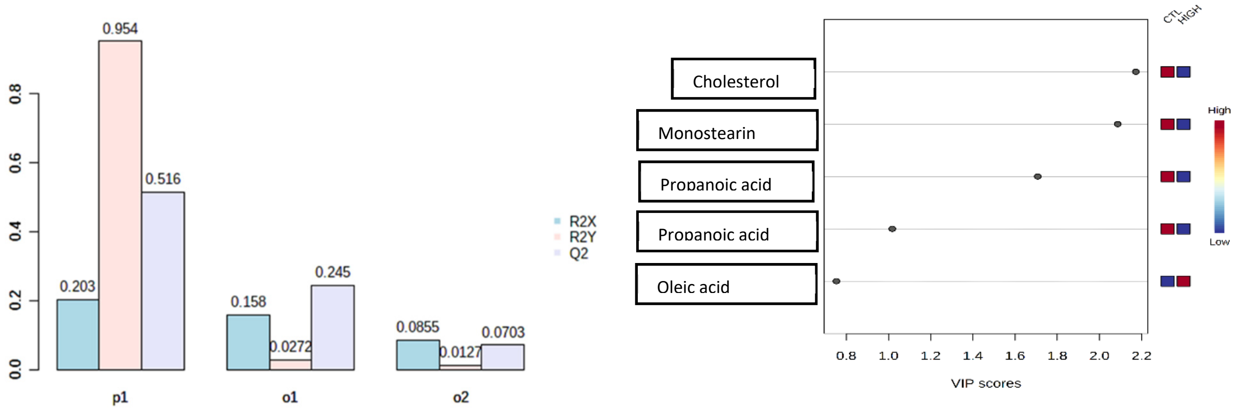

Figure S17. High PFHxS concentration Orthogonal Partial Least Squares - Discriminant Analysis (OPLS-DA) model overview and Variable Importance for Projection (VIP).
